# Psychiatric liaison referrals: a thematic analysis during peak COVID-19

**DOI:** 10.1192/bjo.2021.780

**Published:** 2021-06-18

**Authors:** Kaj Svedberg, William Hancox, Hugh Grant-Peterkin

**Affiliations:** City and hackney Centre for Mental Health

## Abstract

**Aims:**

With the advent of the COVID-19 Pandemic the NHS long term Plan commitments of January 2019 to improve crisis care nationwide became all the more pressing. The aim of this study was to thematically investigate what mental health crisis presentations might be diverted from the Emergency department to external crisis hubs in order to reduce the COVID-19 contamination risks.

**Method:**

All referrals made to the Homerton University Hospital (HUH) mental health liaison service were looked at between 1/3/20-11/6/20 (n = 846), coinciding with the first peak of the COVID-19 Pandemic.

Referral data was anonymised and sorted independently into naturally emerging thematic classes by two junior liaison doctors.

Cases that did not clearly fit any of the 14 themes generated were further looked into to determine outcome of referral and discussed to try and match to an appropriate class.

**Result:**

14 frequent themes for mental health crisis referrals were identified. The distribution of these ranged from most common (suicidality) to neurocognitive presentation and identified shifts in themes over the course of the pandemic peak such as increases of low mood, anxiety and intoxication requiring medical attention over the three month period.

**Conclusion:**

Although themes for presentations may be identified in acute referrals to mental health liaison services it is problematic determining how these may be parsed safely to crisis hubs without risking overlooking cases that may require medical attention. The most common theme that was identified and remained throughout the first wave of the COVID-19 Pandemic was acute suicidal presentation. The remaining themes would require careful consideration around risk thresholds for what a service may wish to accept in devolving the emergency department liaison and balance these against future risks of repeat COVID-19 waves.

